# Evaluation of sorafenib treatment and hepatic arterial infusion chemotherapy for advanced hepatocellular carcinoma: a comparative study using the propensity score matching method

**DOI:** 10.1002/cam4.476

**Published:** 2015-06-04

**Authors:** Kotaro Fukubayashi, Motohiko Tanaka, Kazuhiro Izumi, Takehisa Watanabe, Satomi Fujie, Takeshi Kawasaki, Yoko Yoshimaru, Masakuni Tateyama, Hiroko Setoyama, Hideaki Naoe, Ken Kikuchi, Yutaka Sasaki

**Affiliations:** 1 Department of Gastroenterology and Hepatology, Graduate School of Medical Sciences, Kumamoto UniversityKumamoto, 860-8556, Japan; 2 Medical Quality Management Center, Kumamoto University HospitalKumamoto, 860-8556, Japan

**Keywords:** Disease progression, hepatic arterial infusion chemotherapy, hepatocellular carcinoma, prognosis, propensity score, sorafenib

## Abstract

While sorafenib (SFN) is the established worldwide standard therapeutic agent for advanced hepatocellular carcinoma (HCC), hepatic arterial infusion chemotherapy (HAIC) is also considered a favorable treatment for some advanced HCCs. This study aimed to evaluate each treatment and provide an optimal therapeutic choice for advanced HCCs. We analyzed 72 patients treated with SFN and 128 patients receiving HAIC. Both treatment groups were analyzed for prognostic and disease progression factors, and matched pair analysis was performed using the propensity score matching method. The preferable status of intrahepatic lesions, that is, no lesions or only a single (<3 cm) intrahepetic lesion, was positively associated with good prognosis and negatively associated with disease progression in the SFN group. Maximum tumor size (>5 cm) and low albumin (≤3.4 g/dL) were poor prognostic and disease progression factors in the HAIC group. Analysis of 53 patients selected from each of the SFN and HAIC groups based on the propensity score matching method showed no significant differences in survival or disease progression between the two matched subgroups. On the other hand, progression-free survival (PFS) in the HAIC-matched subgroup was significantly longer than in the SFN-matched subgroup, particularly in patients with portal vein invasion (PVI) and/or without extrahepatic spread (EHS). The treatment efficacy of HAIC is similar to that of SFN regarding survival and disease progression. Longer PFS might be expected for HAIC compared with SFN, particularly in patients with PVI and/or without EHS.

## Introduction

Hepatocellular carcinoma (HCC) is the fifth most commonly diagnosed cancer in the world 1. Recent advances in treatment modalities for HCCs, including surgical resection, liver transplantation, radiofrequency ablation (RFA), and transcatheter arterial chemoembolization (TACE), have improved the clinical course of HCC patients. However, overall prognosis for HCC patients remains poor, as many of these patients are diagnosed at an advanced stage when curative therapies are not applicable. Additionally, even if curative surgical resection or RFA is performed as an initial therapy, the intrahepatic recurrence rate of HCC is high 2,3. Sorafenib (SFN) is a multikinase inhibitor that targets Raf kinase, which participates in tumor cell proliferation, and both vascular endothelial growth factor (VEGF) receptor -2/-3 and platelet-derived growth factor (PDGF) receptor *β*, which contribute to angiogenesis 4. After two randomized controlled studies demonstrated the effectiveness and ability of SFN to prolong both overall survival (OS) and time-to-progression (TTP) 5,6, SFN was approved for use in treating advanced HCC in Japan beginning in May 2009. Subsequently, the Barcelona Clinic Liver Cancer (BCLC) Study Group proposed that SFN might be designated as the standard therapeutic agent in patients with portal vein invasion (PVI) and/or extrahepatic spread (EHS) (BCLC stage C, that is, advanced stage HCC) 7. Although the application of SFN within the clinical setting has been extended over the past few years, clinical improvements in OS and TTP associated with SFN treatment have only been modest 8.

In Japan, hepatic arterial infusion chemotherapy (HAIC) is often performed in HCC patients with vascular invasion and/or refractory status to TACE. In fact, several studies have reported that the HAIC response rate was approximately 20–50%, with a greatly improved prognosis observed in the responders 9–13. However, as there has been a lack of large-scale randomized controlled studies, there is less robust evidence for a survival benefit of HAIC in advanced HCC patients.

Although there have been two comparative cohort studies that examined SFN treatment and HAIC 14,15, a proper comparison of the effectiveness of SFN and HAIC has been difficult, as the patient backgrounds differed between the SFN and the HAIC groups in these studies. In observational studies where the treatment assignment is not random, covariates of the patients receiving one treatment will often differ from covariates of patients receiving a different treatment. It is known that these covariant imbalances can lead to biased treatments that ultimately affect the overall estimates. Within this context, the propensity score matching method has been used to reduce the bias induced by this incomplete matching 16. Observational study designs based on estimated propensity scores can estimate an approximately unbiased treatment effect 17. As SFN has been established as a standard therapeutic agent for advanced HCCs, we evaluated the outcomes of SFN treatment and HAIC, and then compared the treatment efficacy of HAIC to SFN based on the propensity score matching method in order to demonstrate an optimal treatment choice for advanced HCC.

## Methods

### Study population

The study population was composed of a cohort of patients with advanced HCC. Patients with progression of HCC, as documented by PVI, EHS, multiple lesions of both lobes and refractory status to TACE, were considered to be unsuitable for surgical resection or nonsurgical treatments, including RFA or TACE and therefore, were treated by chemotherapy. In this regard, each patient was treated with SFN or HAIC as an initial chemotherapy for advanced HCC.

The patients were diagnosed based on radiological findings of contrast-enhanced computed tomography (CT) and/or magnetic resonance imaging (MRI). Patients treated with SFN were enrolled starting in May 2009, whereas patients who received HAIC were enrolled starting in April 2004, with observation continuing until October 2014 in the Department of Gastroenterology and Hepatology, Kumamoto University Hospital. Eligibility criteria included an Eastern Cooperative Oncology Group (ECOG) performance status score of 2 or less 18, Child–Pugh class A or B, and a life expectancy of at least 12 weeks. Patients with uncontrollable ascites or hepatic encephalopathy were excluded.

This study was conducted with the approval of the Ethics Committee of Kumamoto University and in accordance with Good Clinical Practice and the Declaration of Helsinki guidelines. All patients provided written informed consent before enrollment in the study.

### Treatment protocol

SFN was administered at the standard daily dose of 800 mg, whereas elderly patients (80 years old or older) were started at a reduced daily dose of 400 or 600 mg.

HAIC was implemented using two 5-fluorouracil (5-FU)-based regimens that utilized a subcutaneously placed infusion system. The protocol of the first regimen used a daily administration of cisplatin (10 mg/body) for 1 h followed by 5-FU (250 mg/body) for 5 h on days 1–5. Days 6 and 7 were designated as the off period. A single-treatment course consisted of 4 weeks of the chemotherapy. The second regimen consisted of the administration of interferon (IFN)-*α* (3 million units/body) subcutaneously on days 1, 3, and 5 of each week, and an intraarterial infusion of 5-FU (500 mg/body) on days 1–14 continuously. One course of the treatment lasted 4 weeks. One hundred two patients were treated by the low-dose cisplatin + 5-FU regimen, whereas 26 were treated by the subcutaneous IFN-*α* + 5-FU regimen of HAIC. All patients continued therapy until occurrence of radiological and/or symptomatic progression of the HCC was determined or intolerable adverse events developed.

### Study assessments

A controlled intrahepatic lesion was defined as the detection of no obvious intrahepatic tumor or only a single nodule less than 3 cm in diameter in the patients with EHS.

With regard to tumor type, we defined the nodular, massive, infiltrative, and diffuse types based on Eggel’s classification and the classification of the Liver Cancer Study Group of Japan 19,20. Massive, infiltrative, or diffuse types were classified as nonnodular. Tumor volume >50% was defined as intrahepatic tumors occupying over 50% of the whole liver volume.

OS was determined from the day when treatment was started until the date of death or the final date of confirmed survival. Progression-free survival (PFS) was defined as the time from the start of treatment until disease progression was noted by radiological evaluation and/or clinical progression. Postprogression survival (PPS) was determined from the day that disease progression was confirmed until the date of death or the final date of confirmed survival.

Radiological assessment was performed at 4–12 weeks from the start of treatment using the modified respo-nse evaluation criteria in solid tumors (mRECIST) guidelines 21. Evaluation of tumor response for extrahepatic tumors was performed using the response evaluation criteria in solid tumors (RECIST) guidelines 22.

### Statistical analyses

The difference of means for continuous variables was compared using Student’s *t*-test. Continuous variables were transformed into categorical variables consisting of two ordinal numbers on the basis of the median value. Categorical variables were compared using Fisher’s exact test. OS, PFS, and PPS were calculated using the Kaplan–Meier method, with the difference between the two groups analyzed using a log-rank test. Prognostic and disease progression factors were evaluated, and the hazard ratio and 95% confidence intervals (CIs) were estimated by multivariate analyses performed using the Cox proportional hazards regression model. All factors showing significance in the univariate analysis were then examined by a multivariate analysis. Two-tailed probabilities were used, and *P* < 0.05 was considered statistically significant, unless otherwise noted.

The propensity score was calculated using a binary logistic regression, which included all available variables on liver function and tumor characteristics. Based on a previous review of the prognosis and staging for HCC, these variables were considered to affect the prognosis of the HCC patients 23. A propensity score that reflected the probability of receiving SFN treatment was assigned to each patient. We randomly matched one-to-one pairs of SFN and HAIC patients using a caliper width of 0.01 and no replacement. The two matched subgroups were then analyzed for OS and PFS. All statistical analyses were performed using SPSS software (SPSS Statistics ver. 22; IBM, Chicago, IL).

## Results

### Patient characteristics

The study enrolled 84 patients treated with SFN and 140 patients treated with HAIC. In order to focus on evaluating treatment efficacy, patients were excluded if their treatment was discontinued within 4 weeks from the start of the treatment. Because 12 patients each in the SFN and HAIC groups were forced to discontinue within 4 weeks of starting treatment, due to adverse events, these patients were considered unsuitable for receiving any chemotherapy. After the exclusions, 72 patients treated with SFN and 128 patients treated with HAIC were analyzed in this study. Patient characteristics are summarized in Table1. The characteristics that significantly differed between the two groups were as follows: age, gender, previous treatment, history of TACE, etiology, Child–Pugh class, ascites, tumor type, tumor volume (>50%), number of tumors (>3), EHS, PVI, and controlled intrahepatic lesion (Table1).

**Table 1:** Baseline clinical and tumor characteristics for each group

Variables	SFN group (*N* = 72)	HAIC group (*N* = 128)	*P*-value
Age (years)^1^	68.9 ± 9.84 (69)	65.5 ± 9.32 (67)	0.018
Gender (male/female)	51 (70.8%)/21 (29.2%)	113 (88.3%)/15 (11.7%)	0.004
Previous treatment (yes)	59 (81.9%)	82 (64.1%)	0.009
History of curative therapies (surgery/RFA [yes])	28 (38.9%)	42 (32.8%)	0.441
History of TACE (yes)	56 (77.8%)	78 (60.9%)	0.019
Etiology (HBV/HCV/others)	15 (20.8%)/36 (50%)/21 (29.2%)	33 (25.8%)/77 (60.2%)/18 (14.1%)	0.020
Child–Pugh (A/B)	61 (84.7%)/11 (15.3%)	79 (61.7%)/49 (38.3%)	0.001
Ascites (present/absent)	4 (5.6%)/68 (94.4%)	31 (24.2%)/97 (75.8%)	0.001
Tumor type (nodular/nonnodular)	50 (69.4%)/22 (30.6%)	59 (46.1%)/69 (53.9%)	0.002
Maximum tumor size (>5 cm yes/no)	28 (38.9%)/44 (61.1%)	66 (51.6%)/62 (48.4%)	0.105
Tumor volume (>50% yes/no)	5 (6.9%)/67 (93.1%)	24 (18.8%)/104 (81.2%)	0.023
Number of tumors (>3 yes/no)	53 (73.6%)/19 (26.4%)	114 (89.1%)/14 (10.9%)	0.009
Extrahepatic spread (present/absent)	33 (45.8%)/39 (54.2%)	33 (25.8%)/95 (74.2%)	0.005
Portal vein invasion (present/absent)	21 (29.2%)/51 (70.8%)	64 (50%)/64 (50%)	0.005
Controlled intrahepatic lesion (yes/no)^2^	11 (15.3%)/61 (84.7%)	1 (0.8%)/127 (99.2%)	<0.001
*α*-fetoprotein (ng/mL)^1^	13462.3 ± 46460.9 (186.8)	18471.9 ± 77176.9, (207.8)	0.616
*α*-fetoprotein-L3 (positive/negative)	48 (67.6%)/23 (32.4%)	73 (78.5%)/20 (21.5%)	0.152
PIVKA-II (mAU/mL)^1^	6132.0 ± 20146.8, (421)	9708.9 ± 22224.1, (1421)	0.265

SFN, sorafenib; HAIC, hepatic arterial infusion chemotherapy; RFA, radiofrequency ablation; TACE, transcatheter arterial chemoembolization; HBV, hepatitis B viral infection; HCV, hepatitis C viral infection; PIVKA-II, protein induced by vitamin K absence or antagonists-II.

1 Continuous variables are given as mean ± SD, (median).

2 Controlled intrahepatic lesion: no obvious intrahepatic tumor or single nodule under 3 cm.

### Treatment efficacy, and prognostic and disease progression factors in the SFN group

In the SFN group, 3 (4.2%), 8 (11.1%), 43 (59.7%), and 18 (25.0%) of the patients exhibited complete response (CR), partial response (PR), stable disease (SD), and progressive disease (PD), respectively, with a response rate of 15.3% (Table2). The median OS was 12.5 months, whereas the median PFS was 3.5 months.

**Table 2:** Therapeutic effect observed in each group

	SFN group (*N* = 72)	HAIC group (*N* = 128)
Complete response (CR)	3 (4.2%)	5 (3.9%)
Partial response (PR)	8 (11.1%)	29 (22.7%)
Stable disease (SD)	43 (59.7%)	53 (41.4%)
Progressive disease (PD)	18 (25%)	41 (32%)
Response rate	11 (15.3%)	34 (26.6%)
Median overall survival (OS)	12.5 months	8.8 months
Median progression-free survival (PFS)	3.5 months	3.9 months

Evaluation of tumor response regarding intrahepatic tumors was performed using the Modified Response Evaluation Criteria In Solid Tumors (mRECIST) guidelines, and evaluation of tumor response regarding extrahepatic tumors was performed using the Response Evaluation Criteria In Solid Tumors (RECIST) guidelines. SFN, sorafenib; HAIC, hepatic arterial infusion chemotherapy.

**Figure 1:**
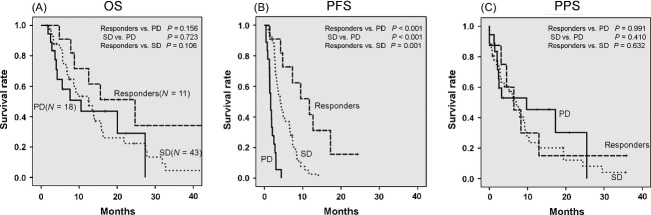
Kaplan–Meier analysis of overall survival (OS), progression-free survival (PFS) and postprogression survival (PPS) for the SFN group. (A) There was no significant difference in the OS among each of the response groups (responders vs. PD; *P *=* *0.156, SD vs. PD; * *=* *0.723, responders vs. SD; *P *=* *0.106). (B) The PFS of the responders and the SD patients was significantly longer than that of the PD patients (responders vs. PD; *P *<* *0.001, SD vs. PD; *P *<* *0.001). In addition, the PFS of the responders was significantly longer than that of the SD patients (*P *=* *0.001). (C) There was no significant difference in the PPS among each of the response groups (responders vs. PD; *P *=* *0.991, SD vs. PD; *P *=* *0.410, responders vs. SD; *P *=* *0.632). SFN, sorafenib; Responders, CR + PR; CR, complete response; PR, partial response; SD, stable disease; PD, progressive disease.

We then performed a Kaplan–Meier analysis to compare the OS, PFS, and PPS of the responders (CR+PR) to the values found for the SD or PD patients. We also compared the OS, PFS, and PPS between the SD and the PD patients. Although we observed no significant differences in the OS and PPS between each of the response groups, we were able to stratify the PFS according to treatment response (Fig.1).

Multivariate analysis of the prognostic factors found that controlled intrahepatic lesion (HR: 0.36; 95% CI: 0.14–0.93; *P *=* *0.034) was significantly associated with good prognosis (Table3).

**Table 3:** Prognostic and disease progression factors in the SFN group as evaluated by the Cox proportional hazards model

	Univariate analysis		Multivariate analysis	
Variables	*P* value	Hazard ratio	95% CI	*P* value
Prognostic factors				
Ascites (present)	0.034	–	–	–
Portal vein invasion	0.038	–	–	–
Controlled intrahepatic lesion^1^	0.028	0.362	0.141–0.926	0.034
Disease progression factors				
Controlled intrahepatic lesion^1^	0.016	0.352	0.171–0.728	0.005
WBC (≤4000/mm^3^)	<0.001	2.835	1.666–4.825	<0.001

SFN, sorafenib; WBC, white blood cell.

1 Controlled intrahepatic lesion: no obvious intrahepatic tumor or single nodule under 3 cm.

Multivariate analysis of the disease progression factors demonstrated that controlled intrahepatic lesion (HR: 0.35; 95% CI: 0.17–0.73; *P *=* *0.005) was negatively associated with disease progression, whereas white blood cell count (≤4000/mm^3^) (HR: 2.84; 95% CI: 1.67–4.83; *P *<* *0.001) was positively associated with disease progression (Table3).

### Treatment efficacy, and prognostic and disease progression factors in the HAIC group

In the HAIC group, 5 (3.9%), 29 (22.7%), 53 (41.4%), and 41 (32.0%) of the patients exhibited CR, PR, SD, and PD, respectively, with a response rate of 26.6%. The median OS was 8.8 months, and the median PFS was 3.9 months (Table2).

**Figure 2:**
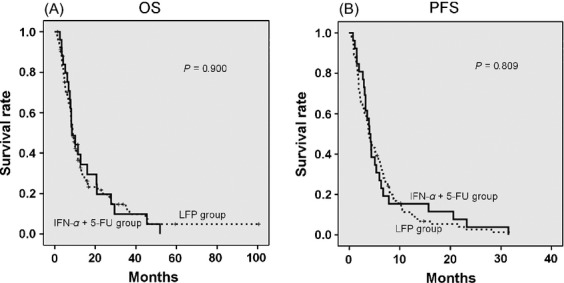
Kaplan–Meier analysis of overall survival (OS) and progression-free survival (PFS) using two different therapeutic regimens in the HAIC group. (A) There was no significant difference in the OS between the LFP group and the IFN-*α *+ 5-FU group (*P *=* *0.900). (B) There was no significant difference in the PFS between the LFP group and the IFN-*α *+ 5-FU group (*P *=* *0.809). HAIC, hepatic arterial infusion chemotherapy; LFP group, patients were treated by low-dose cisplatin + 5-FU hepatic arterial infusion chemotherapy; IFN-*α *+ 5-FU group, patients were treated by subcutaneous interferon-*α *+ 5-FU hepatic arterial infusion chemotherapy.

No significant difference was observed for the OS and PFS between the two therapeutic regimens in the HAIC group (Fig.2).

**Figure 3:**
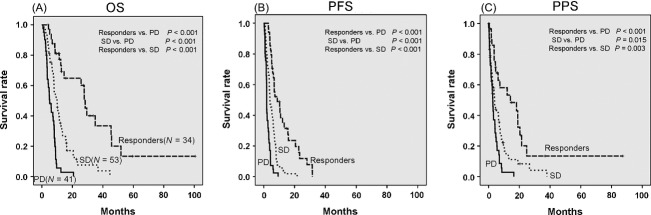
Kaplan–Meier analysis of overall survival (OS), progression-free survival (PFS) and postprogression survival (PPS) for the HAIC group. (A) The OS of the responders and the SD patients was significantly longer than that of the PD patients (responders vs. PD; *P *<* *0.001, SD vs. PD; *P *<* *0.001). In addition, the OS of the responders was significantly longer than that of the SD patients (*P *<* *0.001). (B) The PFS of the responders and the SD patients was significantly longer than that of the PD patients (responders vs. PD; *P *<* *0.001, SD vs. PD; *P *<* *0.001). In addition, the PFS of the responders was significantly longer than that of the SD patients (*P *<* *0.001). (C) The PPS of the responders and the SD patients was significantly longer than that of the PD patients (responders vs. PD; *P *<* *0.001, SD vs. PD; *P *=* *0.015). In addition, the PPS of the responders was significantly longer than that of the SD patients in the HAIC group (*P *=* *0.003). HAIC, hepatic arterial infusion chemotherapy; Responders, CR + PR; CR, complete response; PR, partial response; SD, stable disease; PD, progressive disease.

Using Kaplan–Meier analysis, we compared the OS, PFS, and PPS of the responders to the values found for the SD or PD patients. We also compared the OS, PFS, and PPS between the SD and the PD patients. The results indicated that the OS, PFS, and PPS of the responders and the SD patients were significantly superior to the values found for the PD patients. Additionally, the OS, PFS, and PPS of the responders were also significantly superior to the values found for the SD patients (Fig.3).

Multivariate analysis of the prognostic factors found that maximum tumor size (>5 cm) (HR: 2.05; 95% CI: 1.36–3.10; *P *=* *0.001) and albumin (≤3.4 g/dL) (HR: 2.10; 95% CI: 1.36–3.26; *P *=* *0.001) were significantly associated with poor prognosis (Table4).

**Table 4:** Prognostic and disease progression factors in the HAIC group as evaluated by the Cox proportional hazards model

	Univariate analysis		Multivariate analysis	
Variables	*P* value	Hazard ratio	95% CI	*P* value
Prognostic factors				
Tumor type (nonnodular)	0.024	–	–	–
Maximum tumor size (>5 cm)	0.008	2.049	1.355–3.096	0.001
Tumor volume (>50%)^1^	0.041	–	–	–
Extrahepatic spread	0.046	–	–	–
Albumin (≤3.4 g/dL)	0.012	2.100	1.355–3.256	0.001
WBC (≤4600/mm^3^)	0.035	–	–	–
Disease progression factors				
Ascites (present)	0.014	–	–	–
Maximum tumor size (>5 cm)	0.004	1.721	1.179–2.513	0.005
Tumor volume (>50%)^1^	0.034	–	–	–
Extrahepatic spread	0.014	1.748	1.153–2.646	0.008
Albumin (≤3.4 g/dL)	0.024	1.873	1.265–2.774	0.002
AST (>67 IU/L)	0.015	–	–	–
ALT (>45 IU/L)	0.042	1.508	1.045–2.174	0.028
ALP (>438 IU/L)	0.035	–	–	–

HAIC, hepatic arterial infusion chemotherapy; WBC, white blood cell; AST, aspartate aminotransferase, ALT, alanine aminotransferase; ALP, alkaline-phosphatase.

1 Tumor volume (>50%): intrahepatic tumors occupied over 50% of the whole liver volume.

Multivariate analysis of disease progression factors revealed that maximum tumor size (>5 cm) (HR: 1.72; 95% CI: 1.18–2.51; *P *=* *0.005), EHS (HR: 1.75; 95% CI: 1.15–2.65; *P *=* *0.008), albumin (≤3.4 g/dL) (HR: 1.87; 95% CI: 1.27–2.77; *P *=* *0.002), and alanine aminotransferase (ALT) (>45 IU/L) (HR: 1.51; 95% CI: 1.05–2.17; *P *=* *0.028) were significantly associated with disease progression in the HAIC group (Table4).

### Comparison of the SFN-matched subgroup with the HAIC-matched subgroup using the propensity score matching method

**Figure 4:**
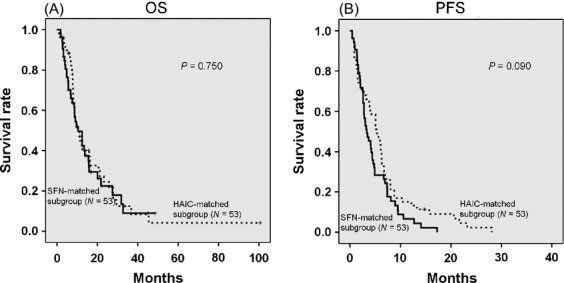
Comparison of the SFN-matched subgroup with the HAIC-matched subgroup using the propensity score matching method (A) Comparison of the overall survival (OS) of the SFN-matched subgroup with that of the HAIC-matched subgroup. There was no significant difference in the OS between the two matched subgroups (*P *=* *0.750). (B) Comparison of the progression-free survival (PFS) between the SFN-matched subgroup and the HAIC-matched subgroup. There was no significant difference in the PFS between the two matched subgroups (*P *=* *0.090). SFN, sorafenib; HAIC, hepatic arterial infusion chemotherapy.

**Figure 5:**
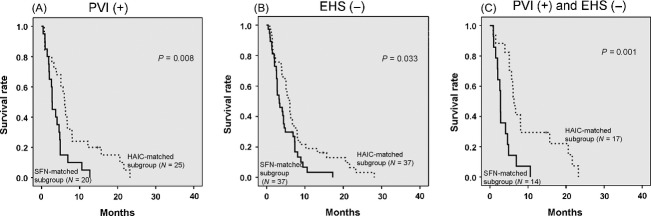
Comparison of progression-free survival (PFS) between both matched subgroups stratified by the presence of portal vein invasion (PVI) and/or the absence of extrahepatic spread (EHS). (A) Comparison of the PFS between both matched subgroups with PVI. The PFS of the HAIC-matched subgroup was significantly longer than that of the SFN-matched subgroup (*P *=* *0.008). (B) Comparison of the PFS between both matched subgroups without EHS. The PFS of the HAIC-matched subgroup was significantly longer than that of the SFN-matched subgroup (*P *=* *0.033). (C) The PFS of the HAIC-matched subgroup was significantly longer than that of the SFN-matched subgroup in the presence of PVI and the absence of EHS (*P *=* *0.001). SFN, sorafenib; HAIC, hepatic arterial infusion chemotherapy.

The propensity score was calculated by performing a binary logistic regression based on liver function and tumor characteristics, which included Child–Pugh class, ascites, tumor type, maximum tumor size (>5 cm), tumor volume (>50%), number of tumors (>3), EHS and PVI. A controlled intrahepatic lesion was determined to be a favorable prognostic factor in the SFN group. On the other hand, a low albumin value was significantly associated with poor prognosis in the HAIC group. As these variables were added to our calculations, the SFN- and HAIC-matched subgroups were created from 53 randomly selected patients of each group that had approximately the same propensity scores. Comparison of liver function and tumor characteristics between the two matched subgroups found no significant differences in these variables (Table5). No significant difference was observed in the OS and the PFS between the two matched subgroups (Fig.4). Furthermore, subanalyses of the matched subgroups were able to stratify the results according to tumor burden (presence or absence of PVI, and EHS). The PFS of the HAIC-matched subgroup was significantly longer than that of the SFN-matched subgroup in the presence of PVI (Fig.5A) and, in addition, was also significantly longer in the absence of EHS (Fig.5B). When the two subgroups were compared when PVI was present and EHS was absent, there was a significantly longer PFS of the HAIC-matched subgroup compared to that of the SFN-matched subgroup (Fig.5C).

**Table 5:** Characteristics of liver function and tumors for each matched subgroup using the propensity score matching method

	SFN-matched subgroup (*N* = 53)	HAIC-matched subgroup (*N* = 53)	*P* value
Child–Pugh (A/B)	42/11	42/11	1.000
Ascites (present/absent)	4/49	4/49	1.000
Albumin (g/dL)^1^	3.475 ± 0.530	3.481 ± 0.533	0.956
Tumor type (nodular/nonnodular)	33/20	33/20	1.000
Maximum tumor size (>5 cm yes/no)	25/28	18/35	0.235
Tumor volume (>50% yes/no)^2^	5/48	9/44	0.390
Number of tumors (>3 yes/no)	45/8	49/4	0.359
Extrahepatic spread (present/absent)	16/37	16/37	1.000
Portal vein invasion (present/absent)	20/33	25/28	0.432
Controlled intrahepatic lesion^3^ (yes/no)	1/52	1/52	1.000

SFN-matched subgroup: patients treated by sorafenib whose baseline characteristics were matched using propensity score matching method. HAIC-matched subgroup: patients treated by low-dose cisplatin + 5-FU hepatic arterial infusion chemotherapy or subcutaneously interferon-*α *+ 5-FU hepatic arterial infusion chemotherapy whose baseline characteristics were matched using propensity score matching method.

1 Continuous variables are given as mean ± SD.

2 Tumor volume (>50%): intrahepatic tumors occupied over 50% of the whole liver volume.

3 Controlled intrahepatic lesion: no obvious intrahepatic tumor or single nodule under 3 cm.

## Discussion

This study demonstrated that OS of the patients in the SFN group could not be stratified, whereas the PFS could be stratified according to treatment response (Fig.1A and B). This finding may be supported by the fact that no significant differences were observed for the PPS between each of the individual response groups (Fig.1C). Previous studies of patients with malignancy of other organs revealed that there was a higher correlation between PPS and OS versus that between PFS and OS 24–26. Additionally, these previous studies also suggested that addressing PPS and subsequent treatments are important factors when evaluating OS 24–26. On the other hand, it has been previously reported that the PPS of HCC patients treated with SFN was influenced by the clinical and tumor status at the time of disease progression 27. Taken together, these findings suggest that clinical and tumor status at the time of progression and/or subsequent treatments may affect PPS and OS to a greater degree than the actual treatment response to SFN.

A controlled intrahepatic lesion was positively associated with a good prognosis, whereas it was negatively associated with disease progression in the SFN group (Table3). This is supported by a previous report describing that a well-controlled intrahepatic tumor was an important prognostic factor in patients with extrahepatic metastasis 28.

In Japan, HAIC is often used to treat patients with advanced HCC 29. The main therapeutic regimens used for HAIC include low-dose cisplatin + 5-FU 9–11, and IFN-*α *+ 5-FU 12,13. The anticancer effects of 5-FU are exerted through the inhibition of thymidylate synthase and the incorporation of its metabolites into the RNA and DNA 30. Because cisplatin enhances 5-FU by inhibiting intracellular l-methionine metabolism, which leads to an increase in the reduced folate pool 31, cisplatin is regarded as a biochemical modulator of 5-FU. With regard to the antitumor effect of IFN-*α*, it has been reported that the induction of p53 by IFN-*α* enhances the apoptotic response to 5-FU, which is correlated with the p53 status of the cancer cells 32. In any case, the key agent for these two therapeutic regimens is 5-FU, which is modulated by cisplatin or IFN. A previous study has reported that the efficacy of the IFN-*α *+ 5-FU regimen was similar to that of best salvage therapy (low-dose cisplatin + 5-FU or cisplatin alone intraarterial infusion chemotherapy) in advanced HCC patients with a high degree of vascular invasion 33. IFN-*α *+ 5-FU therapy exerted modest antitumor effects and posed no safety concerns for patients with advanced HCC. This combination therapy was recommended to be established as a treatment choice for patients with highly advanced HCC 33. In this study, the efficacy of low-dose cisplatin + 5-FU regimen was similar to that of the IFN-*α *+ 5-FU regimen (Fig.2); consequently, we evaluated HAIC using two different regimens.

The response rate of the HAIC group in this study was 26.6% (Table2), which was relatively low compared with the rate reported in previous studies 9–13. It should be noted that while almost all of the previous studies excluded patients with EHS from the eligible criteria, this study included patients with EHS (25.8%, Table1). This might account, in part, for the difference in the response rate to HAIC observed between the current and previous studies.

In this study, we were able to stratify OS, PFS, and PPS in the HAIC group according to the treatment response (Fig.3). Once patients began to respond to HAIC, the tumor status in some of these patients (17 of the 31 responders) was better at the time of progression than at the point where HAIC was first started. Based on these results, it can be speculated that the treatment response may have strongly affected OS and PPS in the HAIC group. When the previous studies 9–13 and this study are taken together, these findings suggest that patients who respond to HAIC will have a good prognosis.

Application of HAIC is performed in a way that ensures the anticancer agents can be administered locally via the hepatic artery, which results in high concentrations in the liver and low concentrations in other organs. In line with this, this study showed that the presence of EHS was a disease progression factor (Table4). These findings suggest that HAIC might be less effective for patients with EHS. However, HAIC should not be excluded as a treatment option for patients having advanced HCC with EHS, because EHS was found to not be a prognostic factor, and six of 33 patients with EHS in this study were responders to HAIC.

Finally, we used the propensity score matching met-hod to compare the effectiveness of SFN and HAIC. The baseline characteristics between the patients in the two treatment groups were statistically different, such as in terms of the tumor-related factors and hepatic functional reserve. However, we recognize that our treatment selection criteria are similar to the criteria for treating advanced HCC, so we compared the outcomes between the two treatments for subclassifying patients according to the estimated propensity score. A previous study that examined HAIC and palliative treatment using the propensity score matching method reported that HAIC appeared to be more useful than palliative treatment 11. To the best of our knowledge, studies using matched baseline characteristics of patients with advanced HCC in comparative cohort studies of SFN and HAIC have yet to be performed. Therefore, this study is the first demonstration to use the propensity score matching method to compare the effects of SFN and HAIC in advanced HCC patients.

The results of our matched comparative study showed that OS and PFS of HAIC were similar to that of SFN (Fig.4). On the contrary, PFS was significantly longer in the HAIC group versus the SFN group in the presence of PVI and/or in the absence of EHS (Fig.5). This suggests that HAIC might be the first treatment choice for advanced HCC patients with PVI and/or without EHS.

There were several limitations for this study. This study did not adopt a randomized design. In addition, it was performed at a single institution, and the overall sample size was small. However, it should be noted that the propensity score matching method was applied in a manner that was able to estimate approximate unbiased treatment effect for both SFN and HAIC in advanced HCC patients.

In summary, the present matched cohort study demonstrated that the treatment efficacy of HAIC was similar to SFN in terms of survival and disease progression, and suggested that HAIC might be advantageous over SFN in terms of PFS, particularly in patients with PVI and/or without EHS.
